# Cytokine Expression of Lung Bacterial Infection in Newly Diagnosed Adult Hematological Malignancies

**DOI:** 10.3389/fimmu.2021.748585

**Published:** 2021-12-02

**Authors:** Zengzheng Li, Zefeng Yang, Peng Hu, Xin Guan, Lihua Zhang, Jinping Zhang, Tonghua Yang, Chaoran Zhang, Renbin Zhao

**Affiliations:** ^1^ Department of Hematology, The First People’s Hospital of Yunnan Province, Kunming, China; ^2^ Yunnan Blood Disease Clinical Medical Center, The First People’s Hospital of Yunnan Province, Kunming, China; ^3^ Yunnan Blood Disease Hospital, The First People’s Hospital of Yunnan Province, Kunming, China; ^4^ Kunming University of Science and Technology School of Medicine, Kunming, China

**Keywords:** IL-6, IL-8, IL-10, hematological malignancy, lung infection, bacterial

## Abstract

Adult patients with hematological malignancies are frequently accompanied by bacterial infections in the lungs when they are first diagnosed. Sputum culture, procalcitonin (PCT), C-reactive protein (CRP), body temperature, and other routinely used assays are not always reliable. Cytokines are frequently abnormally produced in adult hematological malignancies associated with a lung infection, it is uncertain if cytokines can predict lung bacterial infections in individuals with hematological malignancies. Therefore, we reviewed 541 adult patients newly diagnosed with hematological malignancies, of which 254 patients had lung bacterial infections and 287 patients had no other clearly diagnosed infections. To explore the predictive value of cytokines for pulmonary bacterial infection in adult patients with hematological malignancies. Our results show that IL-4, IL-6, IL-8, IL-10, IL-12P70, IL-1β, IL-2, IFN-γ, TNF-α, TNF-β and IL-17A are in the lungs The expression level of bacterially infected individuals was higher than that of patients without any infections (P<0.05). Furthermore, we found that 88.89% (200/225) of patients with IL-6 ≥34.12 pg/ml had a bacterial infection in their lungs. With the level of IL-8 ≥16.35 pg/ml, 71.67% (210/293) of patients were infected. While 66.10% (193/292) of patients had lung bacterial infections with the level of IL-10 ≥5.62 pg/ml. When IL-6, IL-8, and IL-10 were both greater than or equal to their Cutoff-value, 98.52% (133/135) of patients had lung bacterial infection. Significantly better than PCT ≥0.11 ng/ml [63.83% (150/235)], body temperature ≥38.5°C [71.24% (62/87)], CRP ≥9.3 mg/L [53.59% (112/209)] the proportion of lung infection. In general. IL-6, IL-8 and IL-10 are abnormally elevated in patients with lung bacterial infections in adult hematological malignancies. Then, the abnormal increase of IL-6, IL-8 and IL-10 should pay close attention to the possible lung bacterial infection in patients.

## Introduction

Respiratory complications, particularly bacterial infections in the lungs, are very common in patients with hematological malignancies. Immunosuppression caused by hematological malignancies can make patients’ respiratory tracts more susceptible to infections. Patients with hematological malignancies must take immediate action if they develop lung infections, as severe infections increase the risk of death, and approximately 16% of patients have a clear correlation with lung infections (1, 2). Body temperature, PCT, CRP, blood culture, sputum culture, and other detection methods are simple to carry out and extensively used for the diagnosis of such infections (2). The PCT examination, which is now an essential clinical approach for predicting bacterial infections, provides a faster result. The most significant limitation of blood culture and sputum culture methods for detecting the presence of infection is that they are slow and may have low sensitivity. Although body temperature is a simple method, the diagnosis needs further confirmation because the abnormal expression of inflammatory factors (pyrogenic cytokine IL-1) and non-infectious causes can both cause the body temperature to rise (3). In addition, in the absence of other examination equipment and technology to determine whether there is a lung infection, clinicians will consider the use of antibiotics after the body temperature rises, which has caused the proliferation of antibiotics in some areas. It may further cause the production of resistant strains. Other testing methods, such as computerized tomography (CT) examination and chest X-ray, can also be used to confirm the diagnosis. However, more skilled technicians are required to identify the results. And may be restricted by health centers, especially CT.

Interleukins are the most important group of cytokines released during infectious processes. They encompass a large variety of proteins secreted by leukocytes and endothelial cells (among others) and that contribute to cell signaling and pro mote activation, proliferation, death, and/or motility of immune cells (4). They are artificially divided into pro- and anti-inflammatory interleukins. Pro-inflammatory interleukins (including IL-1, Tumor necrosis factor(TNF), Interferon(IFN)-γ and IL-6) are supposed to be responsible for cell activation, tissue damage, and necrosis while anti-inflammatory interleukins (such as IL-4, IL-10, IL-13, IFN-α and Transforming growth factor(TGF)-β) aim to dampen and finally reverse the inflammatory process (5, 6). A lack of balance between pro-inflammatory and anti-inflammatory cytokines might cause the immune system to lose its normal function (7). The study of Th1/Th2 cytokines in infection has gotten a lot of interest in recent years. IL-6 and IL-8 are being researched more in children with hematological malignancies (8–10). There is a considerable association between the rise in IL-6 and PCT, which suggests that the rise in IL-6 may represent the patient’s infection to some extent (11). To identify cytokine indicators such as IL-2, IL-6, IL-8, IL-10, and TNF-α, clinical immunoassay techniques based on ELISA and flow cytometry (CBA) have been developed. The advantages of CBA technology include a wide detection range, ease of operation, minimal sample volume, high sensitivity, and quick operating time (12). This approach is now being used in a large number of studies to predict cytokine expression levels in lung infections in patients with hematological malignancies. It is well known that IL-6 increase can be utilized as a biomarker in children-acquired pneumonia (CAP) (13, 14). Although such differential diagnosis has gained clinical importance, it is currently only used in the investigation of lung bacterial infections in hematological malignancies in children.

Therefore, we reviewed the data of Th1/Th2/Th17 cytokines in 541 newly diagnosed adult patients with hematological malignancies in the Department of Hematology of the First People’s Hospital of Yunnan Province (data from patients who have not received any treatment). The cytokines were compared with PCT, CRP, and body temperature at the same time to investigate the particular cytokine markers in adult patients with hematological malignancies who have lung bacterial infections.

## Materials and Methods

### Patients and Data Collection

From December 2017 to December 2020, a retrospective examination of 541 adult patients with hematological malignancies who did not get any treatment (including chemotherapy and antibiotics) was conducted at the Department of Hematology, the First People’s Hospital of Yunnan Province. In our cohort, viral infections, fungal infections and other infections were not included in our cohort. Only patients with a clear diagnosis of lung bacterial infection(n=254) and patients without any infection were included(n=287). First, record the body temperature, PCT, CRP, Th1/Th2/Th17 cytokines, hemoglobin, white blood cell count, and neutrophil count of the patient during the first examination. The diagnostic criteria for bacterial pulmonary infection were also included (2, 15–17): Regardless of the presence or absence of bacterial cells in respiratory specimens, suspected or proven bacterial lung infections were characterized according to clinical and laboratory guidelines. New or worsening cough, dyspnea, shortness of breath, or hypoxia were the clinical criteria. The laboratory diagnosis included a chest X-ray revealing inflammatory changes and pathogen detection in sputum culture. Due to the limitation of the health center, clinicians prefer to use sputum culture, chest X-ray and clinical standards to determine whether there is bacterial infection in the lungs. After determining the inflammatory changes, CT can no longer be used to check the patient. Therefore, only some patients use CT when they have not been clearly diagnosed with a bacterial infection in the lungs, but when they are suspected of having an infection. The “Helsinki Declaration” and the “International Code of Ethics for Biomedical Research Involving Humans” jointly formulated by the World Health Organization and the Council of International Medical Science Organizations and the relevant regulations of the Ethics Committee of the First People’s Hospital of Yunnan Province were strictly followed during this research. Informed consent of all patients was obtained at the same time.

### Cytokine Determination

The Aimplex Cytokine (QuantoBio, Tianjing, China) was used to measure the serum cytokines levels (Th1/Th2/Th17) including Interferon (IFN)-γ, Interleukin (IL)-1β, IL-2, IL-4, IL-5, IL-6, IL-8, IL-10, IL-12p70, IL-17A, IL-17F, IL-22, Tumor necrosis factor (TNF)-α and TNF-β with the detection range of 1-2500 pg/mL.

### Statistical Analysis

The data were first subjected to a normality test, after which the student’s t-test was used to compare two groups of data belonging to the normal distribution, and the Mann-Whitney U test is used to compare the two sets of data belonging to the non-normal distribution. Interquartile range and median were used to describe continuous data, while percentage and frequency were used to describe categorical variables. The parameters’ optimal Cut-off value was determined using receiver operating characteristics (ROC). IBM SPSS 21.0 (US SPSS) was used for statistical calculations, while GraphPad Prism 7 was used to construct the distribution map. Statistically significant differences were defined as P<0.05.

## Results

### Patient Characteristics

**Figure 1:**
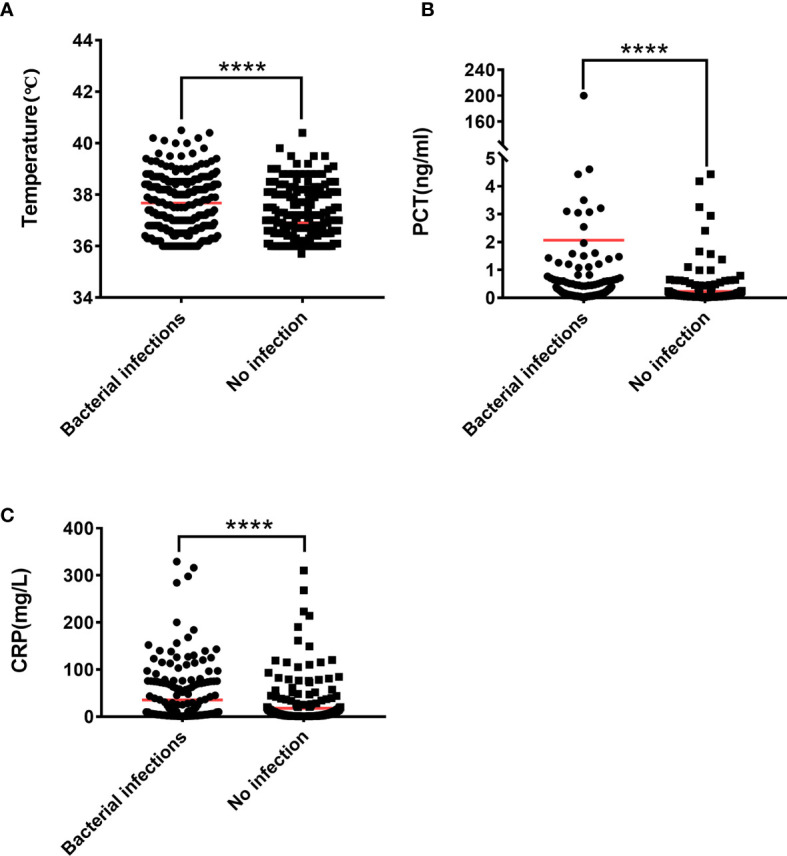
The Mann-Whitney U test is used to test the difference between patients with lung bacterial infection and those without bacterial infection. **(A)** Body temperature, **(B)** PCT, **(C)** CRP. ****P < 0.0001.

A total of 541 untreated (Including chemotherapy and antibiotics) adult patients with newly hematological malignancies were included in our cohort, with 47% (254/541) of those having lung bacterial infections. There were 176 acute myeloid leukemia (AML) patients in the cohort, with 94 males and 82 females, aged 45 (32–58) years old. Lung bacteria were found in 41.48% (73/176) of patients who did not have bacterial infections in their lungs. Patients with bacterial infections of the lungs accounted for 58.52% (103/176). There were 56 multiple myelomas (MM) patients (34 males, and 22 females, aged 56 (51–65) years old) with 62.50% (35/56) of patients with no lung bacterial infection and 37.5% of patients with lung bacterial infection (21/56). There were 29 patients (8 males, 21 females, aged 54 (51-65) years old) with myelodysplastic syndrome (MDS) with 58.62% (17/29) of patients without lung bacterial infection and 41.38% (12/29) patients with lung bacterial infection. There were 135 lymphoma patients (76 males, 59 females, aged 53 (37.00-62.00) years old) with 64.44% (87/135) patients with no lung bacterial infection, and 35.56% (48/135) patients with lung bacterial infection. There were 41 patients (26 men and 15 women, aged 48 (31.50-57.00) years old) with chronic myelogenous leukemia (CML). Patients who did not have a bacterial infection in their lungs made up 60.98% (25/41) of the total, while those who had a bacterial infection in their lungs comprised 39.2% (16/41). There were 51 patients (30 males, 21 females, aged 50 (25.00-75.00) years old) with acute lymphoblastic leukemia (ALL), 60.78% (31/51) of whom were free of lung bacterial infection, and 39.22% (20/51) of whom had lung bacterial infection. There were 53 patients (26 males, 27 females, aged 50 (25-75) years old) with other hematological malignancies, 35.85% (19/53) patients without lung bacterial infection, and patients with lung bacterial infection accounted for 64.15% (34/53) (**Figure 1** and **Table 1**). In our study, 32.68% (83/254) of patients with lung infection were positive for sputum culture. Among them, *Klebsiella pneumoniae* accounted for 24.01% (20/83), *Streptococcus pneumoniae* accounted for 15.67% (13/83), *Pseudomonas aeruginosa* accounted for 14.46% (12/83), *Escherichia coli* accounted for 14.46% (12/83), *Staphylococcus aureus* accounted for 12.04% (10/83), *Haemophilus influenzae* accounted for 8.43% (7/83), Others accounted for 10.93% (9/83) (including *Serratia marcescens*, *maltophilus narrow food list Bacteria*, *Comamonas testosterone*, *Serratia liquefaction* and *Acinetobacter baumannii*).

**Table 1:** Basic characteristics of patients.

Parameters	Gender: (Male/Female)	Age (years)	No bacterial infection in the lungs (n = 287)	Bacterial Lung Infection (n = 254)	P
AML (n=176)	94/82	45 (32-58)	73	103	—
MM (n=56)	34/22	56 (51-64.75)	35	21	—
MDS (n=29)	21/8	54 (43-68)	17	12	—
Lymphoma (n=135)	76/59	53 (37-62)	87	48	—
CML (n=41)	26/15	48 (31.5-57)	25	16	—
ALL (n=51)	30/21	50 (25-75)	31	20	—
Other (n=53)	26/27	50 (25-75)	19	34	—
Temperature (°C)	—	—	36.8 (36.4-37.7)	37.8 (36.7-38.4)	0.000
WBC (×10^9^/L)	—	—	5.74 (2.97-11.06)	4.35 (1.13-11.53)	0.002
PLT (×10^9^/L)	—	—	134 (37-234)	87.5 (37.5-186)	0.029
HGB (g/L)	—	—	104 (80-130)	85 (70-108)	0.000
PCT (ng/ml)	—	—	0.07 (0.04-0.14)	0.16 (0.07-0.42)	0.000
CRP (mg/L)	—	—	4.24 (1.32-13.9)	9.38 (2.98-56.7)	0.000

The composition type of the patient’s disease,and the difference in Temperature, WBC, PLT, HGB, PCT and CRP between the patient’s lung bacterial infection and no infection was detected by the Mann-Whitney U test.

### Difference Analysis of Cytokines

**Figure 2:**
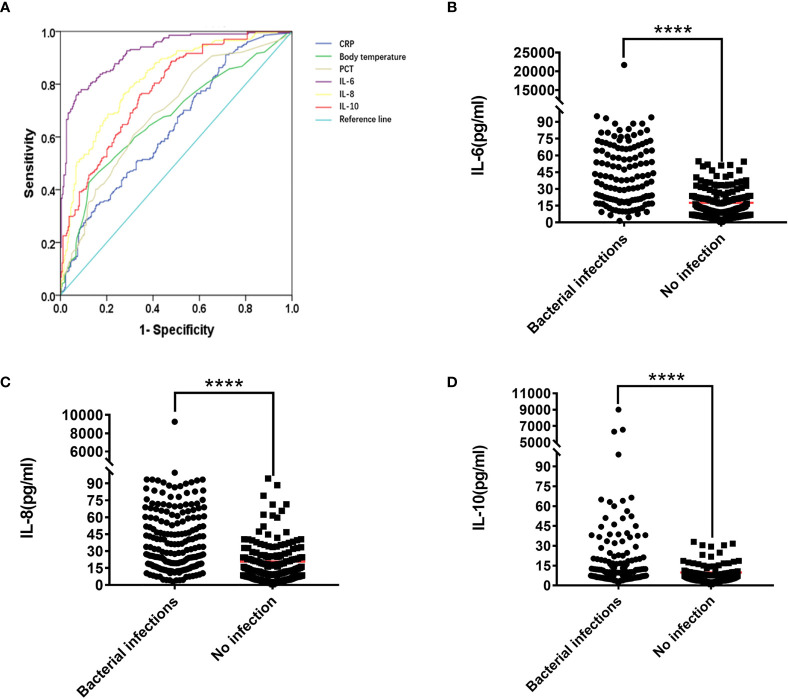
**(A)** ROC curve of IL-6, IL-8, IL-10, PCT, CRP, Temperature. The Mann-Whitney U test was used to test the difference in expression between patients with lung bacterial infection and those without bacterial infection **(B)** IL-6, **(C)** IL-8. **(D)** IL-10. ****P < 0.0001.

The expression levels of cytokines in patients with lung bacterial infection are IL-4: 3.09 (1.80-5.53 pg/ml, IL-5: 2.17 (1.58-3.18) pg/ml, IL-6: 103.90 (38.46-277.95) pg/ml, IL-8: 43.53 (19.20-88.50) pg/ml, IL-10: 8.05 (5.65-18.80) pg/ml, IL-12P70: 5.22 (4.02-6.46) pg/ml, IL-1β: 1.88 (1.36-2.52) pg/ml, IL-2: 3.87 (1.95-4.97) pg/ml, IFN-γ: 3.26 (2.50-4.74) pg/ml, TNF-α: 3.09 (2.24-4.21) pg/ml, TNF-β: 3.39 (2.72-4.33) pg/ml, IL-17A: 2.25 (1.38-3.22) pg/ml, IL-17F: 3.60 (2.59-4.89) pg/ml, IL-22: 1.02 (0.48-1.93) pg/ml. The expression levels of cytokines in patients without lung bacterial infection were IL-4: 2.52 (1.52-3.84) pg/ml, IL-5: 2.13 (1.58-2.74) pg/ml, IL-6: 9 (5.43-19.2) pg/ml, IL-8: 10.43 (5.48-21.04) pg/ml, IL-10: 4.43 (3.36-6.98) pg/ml, IL-12P70: 4.51 (3.34-5.99) pg/ml, IL-1β: 1.68 (1.21-2.13) pg/ml, IL-2: 3.08 (1.96-4.62) pg/ml, IFN-γ: 2.79 (1.98-3.75) pg/ml, TNF-α: 2.81 (1.95-3.65) pg/ml, TNF-β: 2.99 (2.18-3.72) pg/ml, IL-17A: 1.87 (1.20-2.82) pg/ml, IL-17F: 3.42 (2.54-4.62) pg/ml, IL-22: 0.86 (0.41-1.54) pg/ml. The Mann-Whitney U test found that most of these cytokines were statistically different between lung bacterial infection and uninfected patients, except for IL-5, IL-17F, and IL-22 (**Figures 2B–D**, **Supplementary Figures 1A–H** and **Table 2**).

**Table 2:** Analysis of the difference between infected and uninfected.

Parameters (pg/ml)	Bacterial Lung Infection (n = 254)	No bacterial infection in the lungs (n = 287)	P
IL-4	3.09 (1.80-5.53)	2.52 (1.52-3.84)	0.001
IL-5	2.17 (1.58-3.18)	2.13 (1.58-2.74)	0.144
IL-6	103.90 (38.46-277.95)	9.00 (5.43-19.20)	0.000
IL-8	43.53 (19.20-88.50)	10.43 (5.48-21.04)	0.000
IL-10	8.05 (5.65-18.80)	4.43 (3.36-6.98)	0.000
IL-12P70	5.22 (4.02-6.46)	4.51 (3.34-5.99)	0.000
IL-1β	1.88 (1.36-2.52)	1.68 (1.21-2.13)	0.000
IL-2	3.87 (1.95-4.97)	3.08 (1.96-4.62)	0.000
IFN-γ	3.26 (2.50-4.74)	2.79 (1.98-3.75)	0.000
TNF-α	3.09 (2.24-4.21)	2.81 (1.95-3.65)	0.000
TNF-β	3.39 (2.72-4.33)	2.99 (2.18-3.72)	0.000
IL-17A	2.25 (1.38-3.22)	1.87 (1.20-2.82)	0.009
IL-17F	3.60 (2.59-4.89)	3.42 (2.54-4.62)	0.216
IL-22	1.02 (0.48-1.93)	0.86 (0.41-1.54)	0.055

The difference in the expression of cytokines between the patient’s lungs with bacterial infection and without infection was detected by the Mann-Whitney U test.

### Determination of Cutt-Off Value

We used ROC to calculate the Cut-off value to see if there were specific cytokine indicators between bacterial infection and non-infection in the lungs. **Table 3** and **Supplementary Table 1** show that IL-6, IL-8, and IL-10 have the best area under the curve (AUC) between the absence of infection and the presence of infection. They are IL-6 AUC =91.30%, IL-8 AUC =81.50%, IL-10 AUC =77.1% (**Figure 2**), and the Cut-off values are IL-6 = 34.12 pg/ml, IL-8 = 16.35 pg/ml, IL-10 = 5.62 pg/ml (**Supplementary Table 1A** and **Table 3**).

**Table 3:** Cut-off value of IL-6, IL-8, IL-10, PCT, CRP and temperature.

Parameters	Cutt-off	Proportion of infections exceeding the Cutt-off value
IL-6	34.12pg/ml	88.89% (200/225)
IL-8	16.35pg/ml	71.67% (210/293)
IL-10	5.62pg/ml	66.10% (193/292)
PCT	0.11ng/ml	63.83% (150/235)
CRP	9.30mg/L	53.59% (112/209)
Temperature	38.5°C	71.24% (62/87)

Cut-off values of IL-6, IL-8, IL-10, PCT, CRP, Temperature are determined by ROC curve.

### Comparison With PCT, CRP, Body Temperature

PCT, CRP, and body temperature are important indicators for evaluating infections in clinical practice. At the same time, we calculated the PCT, CRP, and body temperature Cut-off values. The results showed that 63.83% (150/235) patients had lung bacterial infection when PCT ≥ 0.11 ng/ml, 71.24% (62/87) patients had lung bacterial infection when body temperature ≥38.5 ℃, and 53.59% (112/209) patients have bacterial lung infections when CRP ≥9.3 mg/L (**Supplementary Table 1** and **Table 3**). Compared with IL-6, IL-8, and IL-10, we found that 88.89% (200/225) of patients with IL-6 ≥34.12 pg/ml had lung bacterial infection, thus indicating a better advantage over PCT, CRP, and body temperature. Patients with IL-8 ≥16.35 pg/ml had a lung bacterial infection in 71.67% (210/293) of cases, which is more accurate than PCT and CRP but nearly equivalent to the accuracy of body temperature over 38.5 ℃. At IL-10 ≥5.62 pg/ml, 66.10% (193/292) of the patients had lung bacterial infection, which was superior to CRP, but not significantly superior to body temperature or PCT. An unexpected finding was that 98.52% (133/135) of patients had lung bacterial infection when the levels of IL-6, IL-8, and IL-10 simultaneously exceeded their critical values.

## Discussion

Clinically, bacterial infection of the lung has always been an important problem, and it affects the treatment strategy and even the course of the disease for patients with hematological malignancies. Abnormalities in the cytokine network produced by bacterial infections in the lungs are common. IL-6 plays a key role in the inflammatory response and is a major immunomodulator (18). It plays an active role in activating T helper cells, inhibiting the differentiation of T regulatory (Tregs) cells and B cells, and has the role of coordinating innate and adaptive immune responses (19). In inflammation, IL-6 binds to membrane-bound IL-6R to form an IL-6-IL-6R complex, which then binds to gp130 (glycoprotein 130) to generate signal transduction through the JAK-STAT pathway (classical signal transduction). IL-6 can also bind to soluble forms of the IL-6R before converging on gp130 (trans-signaling), or be trans-presented from dendritic cells *via* their membrane-bound IL-6R to T cells (trans-presentation). In general terms, classical signaling causes the physiological, anti-inflammatory, and pro-resolution effects of IL-6, with the pathological effects of the cytokine mediated by trans-signalling and trans-presentation (20). The plasma concentration of IL-6 in healthy adults ranges from 0.2 to 7.8 pg/mL, but the concentration in children ranges from 18 to 26 pg/mL (21–23). However, IL-6 concentrations can exceed 1600 pg/mL in patients with sepsis (24). The level of IL-6 in the blood has also been shown in literature search and analysis studies to have clinical usefulness in distinguishing people with severe infections (25). Like IL-6, the expression of IL-8, which has a pro-inflammatory effect, is stimulated by a variety of factors (including IL-6 and bacterial particles) (20, 26). IL-8 mediates its signal by extracellularly binding to two G protein-coupled receptors, CXC chemokine receptor 1 (CXCR1) and CXC chemokine receptor 2 (CXCR2). The CXCL8-CXCR1/2 axis recruits neutrophils at the site of infection and induces a neutrophil oxidative burst and a granule release to eliminate inflammatory stimulus and increase bacterial clearance (27, 28). Since CXCR2 is more sensitive to low ligand concentrations, CXCR2 is believed to play a more important role at recruiting neutrophils to the site of infection (including bacterial infections in the lungs), whereas CXCR1 mediates oxidative burst and granule release to combat the microbes at the site of infection (29). IL-6 and IL-8 levels are elevated not just in children with hematological malignancies, but also when there is an invasive fungal infection of the lungs in children with hematological malignancies and may signal the patients’ early death (30).

IL-10 is a well-known anti-inflammatory cytokine that can be produced by almost all immune cells and can help to maintain and restore immunological homeostasis (31, 32). Immunosuppressive properties of IL-10 are pleiotropic. By suppressing the pro-inflammatory response caused by various immune cells, IL-10 limits tissue damage and immunopathology caused during infection (31, 33, 34). During pneumococcal pneumonia in mice, high levels of IL-6, IL-1β and TNF-α are produced in the early stages of lung parenchyma and/or bronchoalveolar space infection. The production of these pro-inflammatory cytokines caused a large influx of neutrophils into the lungs of infected mice and increased tissue damage 24 hours after infection. However, an increase in IL-10 expression and a decrease in the number of neutrophils were observed 48 hours after infection (35). In addition to reducing the number of pro-inflammatory cytokines, IL-10 may also play a role in regulating the recruitment of neutrophils and the spread of bacteria from the lungs to other tissues (36).Our previous research found that an abnormal increase in IL-10 in the secondary hemophagocytic syndrome of the cytokine storm could predict the patient’s death (37). These findings indicate that IL-10 is abnormally expressed after the patient is infected. In adult patients with hematological malignancies with bacterial infection of the lungs, as an anti-inflammatory factor, the increase of IL-10 may be to inhibit the high expression of the pro-inflammatory factors IL-6 and IL-8. What needs to be explained here is that the malignant hematological disease itself may have immunosuppression in untreated patients, and cytokines may also be disordered after immunosuppression. The complex relationship among immunosuppression, infection and cytokine expression still needs to be studied in depth.

In our study, only adult patients with hematological malignancies were studied. The main reason is that we only accept patients with hematological malignancies in our daily diagnosis and treatment. Other studies reported that in the study of childhood hematological malignancies, the increase in the expression levels of IL-6 and IL-10 predicted the occurrence of infection in patients (1, 38), and the expression level of children with bacteremia was significantly higher than Children infected with the virus (1). In addition, IL-10 are also abnormally elevated in pneumonia after esophageal cancer surgery (39). Abnormal cytokines in infection are common in studies of non-tumor patients. IL-6, IL-8, and IL-10 are significantly abnormally expressed in acquired pneumonia (whether children or adult patients) (40–43). The expression of IL-6 and IL-10 in the serum of patients with enterovirus/bacterial meningitis is abnormal, while the expression of IL-6 and IL-10 in patients with bacterial meningitis is higher than that of viral meningitis (44). In 2019 coronavirus disease (COVID-19), severe acute respiratory syndrome (SARS) and Middle East respiratory syndrome (MERS), the abnormal increase in IL-6 is related to the severity of the disease (45, 46). IL-6 and IL-8 will also increase in patients with fungal infections such as *Candida* (47–49). IL-10 is also abnormally elevated in herpes simplex virus type I infection (50), but it is not abnormally expressed in influenza A, influenza B, and influenza-like diseases (51). In patients with periodontitis, LI-6 and IL-8 are abnormally elevated, and IL-6 in the cerebrospinal fluid after subarachnoid hemorrhage can predict infection (27, 30, 52). This seems to indicate that the increase in IL-6, IL-8 and IL-10 is a common result of infection. Other factors such as smoking, viruses, and a person’s disease can cause the increase of IL-6, IL-8 and IL-10 in the patient’s body (30, 53–55)., Our research cannot completely rule out the influence of these factors and other undiagnosed infections. These evidences seem to indicate that elevated IL-6, IL-8 and IL-10 in non-tumor patients are common results of infections (including infections of different sites and different microorganisms), so our data does not support bacterial infections in the lungs The specificity. Anti-infective treatment should be combined with underlying diseases, clinical symptoms and other test result.

The Cut-off values for IL-6, IL-8, and IL-10 reported by different researchers differ slightly (1, 10, 56, 57). Their research, as well as ours, have revealed the benefits of cytokines IL-6, IL-8, and IL-10 in accurately predicting infections with a short detection time and high sensitivity. It should be noted that the abnormal increase of IL-6, IL-8 and IL-10 in patients with hematological malignancies may not only occur when bacterial infections in the lungs occur. It may also occur when infection occurs in other parts, and when other microorganisms are infected. Therefore, when IL-6, IL-8 and IL-10 are found to be abnormally elevated, clinical symptoms and other examination results should be combined to identify the type of infectious agent, which will facilitate the choice of anti-infective drugs. In view of other people’s research and our results, we believe that cytokines, especially IL-6, IL-8 and IL-10, should be tested in patients with hematological malignancies. In this way, we may be able to use cytokines to help determine whether a patient has an infection. In addition, sometimes the newly diagnosed patients do not have obvious characteristics of lung infection (such as fever, positive sputum culture, cough, dyspnea, elevated PCT and CRP, etc.), and it is easy to ignore lung bacterial infection. Then, when there are no other clear laboratory indicators to indicate infection, one should be alert to the abnormal increase of IL-6, IL-8 and IL-10, which may indicate bacterial infection in the lungs. Our study is a retrospective single-center study with the following limitations: 1. The research of different types of diseases is not carried out separately, and other tumors are not included for comparison, so our data cannot support the specificity in hematological malignancies; 2. The data of fungi and viruses are not included, so our data does not support the specificity of IL-6, IL-8 and IL-10 in bacteria; 3. Since we only reviewed patients with bacterial infections in the lungs, our data may not support the specificity of the lungs. Our next study will focus on these deficiencies and the development of IL-6, IL-8, and IL-10 as separate tests for the diagnosis of adult hematological malignant pulmonary bacterial infections.

## Conclusion

In summary, our data indicate that IL-6, IL-8, and IL-10 are abnormally elevated in patients with lung bacterial infections in adult hematological malignancies. At the same time, the simultaneous increase of IL-6, IL-8 and IL-10 has a better AUC than PCT/CRP/Temperature. Then, the abnormal increase of IL-6, IL-8 and IL-10 should pay close attention to the possible lung bacterial infection in patients.

## Data Availability Statement

The original contributions presented in the study are included in the article/**Supplementary Material**. Further inquiries can be directed to the corresponding authors.

## Ethics Statement

The studies involving human participants were reviewed and approved by the Ethics Committee of the First People’s Hospital of Yunnan Province. The patients/participants provided their written informed consent to participate in this study.

## Author Contributions

All authors participated in the research and agreed to the publishing license. ZL analyzed the data and drafted the manuscript. ZY reviewed the analysis results and graphs. TY, RZ, and CZ designed the research plan, reviewed the manuscript and revised the main content. PH, XG, LZ, and JZ performed statistics.

## Funding

This work was supported by the National Natural Science Foundation of China (82060810) and Open Project of Yunnan Blood Clinical Medical Center (2019LCZXKF-XY11, 2019LCZXKF-XY09, 2020LCZXKF-XY07 and 2021LCZXXF-XY09).

## Conflict of Interest

The authors declare that the research was conducted in the absence of any commercial or financial relationships that could be construed as a potential conflict of interest.

## Publisher’s Note

All claims expressed in this article are solely those of the authors and do not necessarily represent those of their affiliated organizations, or those of the publisher, the editors and the reviewers. Any product that may be evaluated in this article, or claim that may be made by its manufacturer, is not guaranteed or endorsed by the publisher.
