# Supplementary material for: Cytokine Expression of Lung Bacterial Infection in Newly Diagnosed Adult Hematological Malignancies
Source: Front Immunol. 2021 Dec 2;12:748585. doi: 10.3389/fimmu.2021.748585 (PMC8674689; doi:10.3389/fimmu.2021.748585)
Supplement: Supplementary file 1 [file DataSheet_1.docx]

Supplementary Figure 1
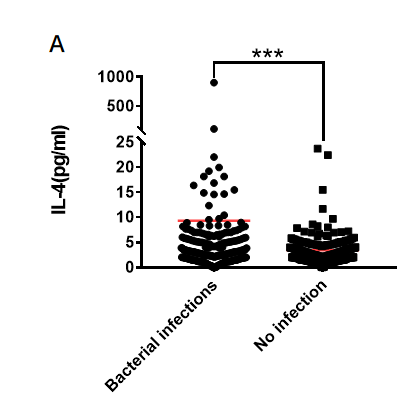

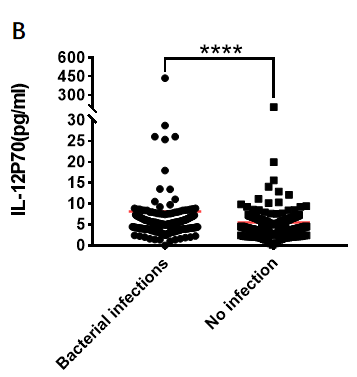

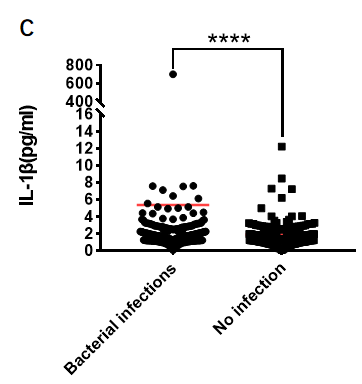

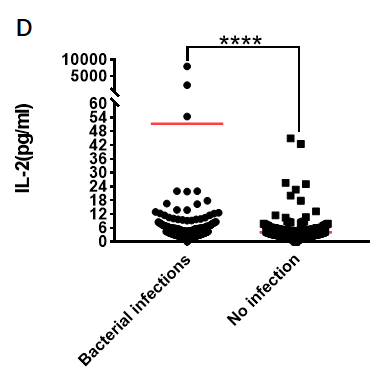

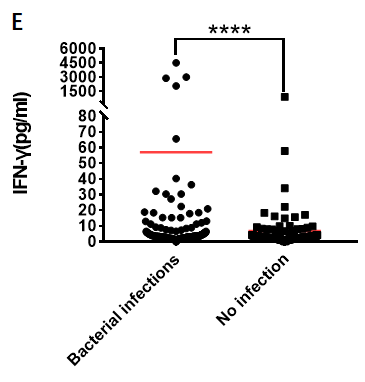

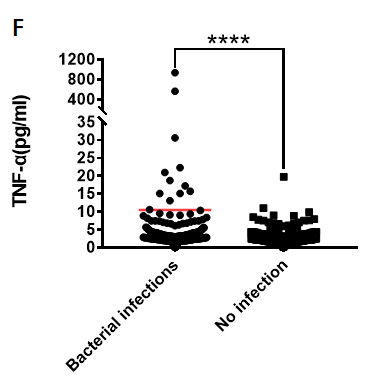


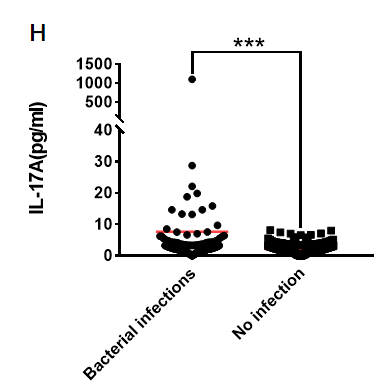


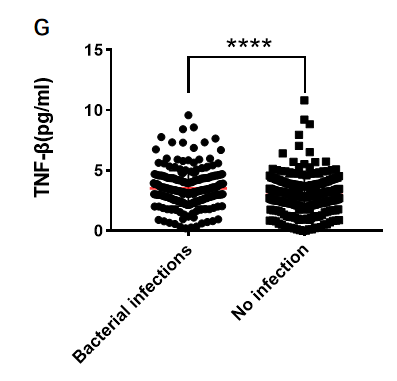


Supplementary Figure 1.The Mann-Whitney U test was used to test the difference in expression between patients with lung bacterial infection and those without bacterial infection A:IL-4,B:IL-12P70,C:IL-1β,D:IL-2,E:IFN-γ,F:TNF-α,G: TNF-β,H:IL-17A. ***:P＜0.001, ****:P＜0.0001.

Supplementary Table 1. AUC of cytokines, CRP, PCT and Temperature

| Parameters | AUC | P | 95% Confidence interval | |
| --- | --- | --- | --- | --- |
|  |  |  | Lower limit | Upper limit |
| IL-4 | 0.574 | 0.01 | 0.517 | 0.631 |
| IL-5 | 0.579 | 0.005 | 0.523 | 0.636 |
| IL-6 | 0.913 | 0.00 | 0.886 | 0.94 |
| IL-8 | 0.815 | 0.00 | 0.775 | 0.856 |
| IL-10 | 0.771 | 0.00 | 0.727 | 0.815 |
| IL-12P70 | 0.591 | 0.001 | 0.537 | 0.646 |
| IL-1β | 0.581 | 0.005 | 0.525 | 0.636 |
| IL-2 | 0.621 | 0.00 | 0.566 | 0.676 |
| IFN-γ | 0.600 | 0.00 | 0.546 | 0.655 |
| TNF-α | 0.564 | 0.025 | 0.508 | 0.619 |
| TNF-β | 0.62 | 0.00 | 0.566 | 0.674 |
| IL-17A | 0.536 | 0.211 | 0.479 | 0.592 |
| IL-17F | 0.524 | 0.395 | 0.468 | 0.581 |
| IL-22 | 0.566 | 0.021 | 0.51 | 0.622 |
| CRP | 0.664 | 0.00 | 0.612 | 0.716 |
| Temperature | 0.657 | 0.00 | 0.603 | 0.711 |
| PCT | 0.686 | 0.00 | 0.635 | 0.737 |

AUC of Cytokines，CRP, PCT, Temperatuer are determined by ROC curve.
